# Genome-wide probabilistic reconciliation analysis across vertebrates

**DOI:** 10.1186/1471-2105-14-S15-S10

**Published:** 2013-10-15

**Authors:** Owais Mahmudi, Joel Sjöstrand, Bengt Sennblad, Jens Lagergren

**Affiliations:** 1 School of Computer Science and Communications, KTH Royal Institute of Technology, Science for Life Laboratory (SciLifeLab), Swedish e-Science Research Centre, Stockholm, Sweden; 2 Department of Numerical Analysis and Computer Science, Stockholm University, Science for Life Laboratory (SciLifeLab), Stockholm, Sweden; 3 Artherosclerosis research unit, Department of Medicine, Karolinska Institute, Science for Life Laboratory (SciLifeLab), Stockholm, Sweden

## Abstract

Gene duplication is considered to be a major driving force in evolution that enables the genome of a species to acquire new functions. A reconciliation - a mapping of gene tree vertices to the edges or vertices of a species tree - explains where gene duplications have occurred on the species tree. In this study, we sample reconciliations from a posterior over reconciliations, gene trees, edge lengths and other parameters, given a species tree and gene sequences. We employ a Bayesian analysis tool, based on the probabilistic model DLRS that integrates gene duplication, gene loss and sequence evolution under a relaxed molecular clock for substitution rates, to obtain this posterior.

By applying these methods, we perform a genome-wide analysis of a nine species dataset, OPTIC, and conclude that for many gene families, the most parsimonious reconciliation (MPR) - a reconciliation that minimizes the number of duplications - is far from the correct explanation of the evolutionary history. For the given dataset, we observe that approximately 19% of the sampled reconciliations are different from MPR. This is in clear contrast with previous estimates, based on simpler models and less realistic assumptions, according to which 98% of the reconciliations can be expected to be identical to MPR. We also generate heatmaps showing where in the species trees duplications have been most frequent during the evolution of these species.

## Introduction

*Phylogenetics *- traditionally a field primarily concerned with inferring tree-like evolution of species - has recently been superseded by *phylogenomics *- which also includes the evolution of genomes and their functional elements, in particular the genes, in relation to the species evolution. This genomics evolution is for many areas of biology, e.g., molecular biology, the final goal, to which the species evolution then is a means. In particular, evolution of genes across species is a result of evolutionary processes such as gene duplication and loss, which in eukaryotes have been shown to be major driving forces in gene evolution.

Goodman et al [1] pioneered the field by introducing the notion of a *reconciliation *of the evolutionary history of a gene family, represented by a gene tree, with that of the corresponding species, represented by a species tree. In general, a reconciliation is a mapping of the gene tree vertices onto the species tree. Each internal vertex of the gene tree is either mapped to (1) a species tree vertex, which implies that the gene tree vertex represents a speciation or (2) a species tree edge, which implies that the gene tree vertex represents a duplication. Goodman et al. used a parsimony approach and gave an algorithm that finds the most parsimonious reconciliation (MPR), i.e., the unique mapping that explains the difference between the gene and species trees using a minimum number of duplications [1].

Arvestad et al. [2] introduced the first probabilistic model for gene evolution, which explains how a gene family evolves inside a species tree by undergoing operations such as gene duplications and gene losses. Later Arvestad et al. [3] proposed an integrated model of gene duplication, gene loss, and sequence evolution, under a molecular clock for estimating the posterior distribution over gene trees. A Markov Chain Monte Carlo (MCMC) based approach was used to get the posterior distribution over gene trees, given sequence data for a gene family and the corresponding species tree. Åkerborg et al. [4] improved the model by introducing a relaxed molecular clock for sequence evolution integrated with gene duplication and gene loss. This framework efficiently computes the posterior over gene trees. Nevertheless, they do not suggest how to obtain reconciliations from the posterior distribution over gene trees. Rasmussen et al. [5] recently introduced another probabilistic approach to reconstruct gene trees inside the species tree. The method uses a hill-climbing-based approach, but it only considers MPR while computing the likelihood of a gene tree. They supported this assumption by a simulation study, where they simulated reconciled gene trees for the species tree using independently estimated duplication and loss rates [6], and found that 98% of all generated reconciliations were identical to MPR. Doyon et al. [7] reported similar results, and concluded that the most likely reconciliation is either identical to MPR or very close to MPR. In a recent study, Doyon et al. [8] using a simple birth-death process and realistic but averaged gene duplication/loss rates, found that a very small subset of all reconciliations needs to be explored in order to approximate the posterior probability of the most likely reconciliations. Åkerborg et al. [4], on the other hand argued that MPR provides an incorrect explanation of the evolutionary history of gene families that have a higher duplication rate.

Recently, genomes of different species have been published with increasingly better coverage. For instance, Heger et al. [9] published the Orthologous and Paralogous Transcripts in Clades (OPTIC) database, which provides sets of gene prediction, gene families, and orthology assignments for clades of amniotes, vertebrates, flies, nematodes and yeasts. In this study, we extend the framework by Åkerborg et al. [4], for computing the posterior over gene trees, by proposing algorithms for sampling reconciliations as well as computing the most likely reconciliations on the vertebrates clade of OPTIC dataset. This allows us to perform a genome-wide study on the OPTIC dataset, in which posteriors over gene trees *and *reconciliations are estimated. We augment the species tree by adding a heatmap for each edge, illustrating how frequently duplications occur on the edge, among the gene families. We also compare the reconciliations we obtain with the most parsimonious and conclude that MPR leads to an incorrect reconciliation in 19% of all reconciliations. Finally, we propose algorithms for sampling and computing the most likely *realizations *(a finer reconciliation, that maps vertices of the gene tree to specific time points on the species tree).

## Methods

In this section, we review the DLRS model and some algorithmic results from [4]. We then continue to show how the latter can be extended so that reconciliation and so-called discretized realizations can be sampled from the posterior distribution, as well as *maximum aposteriori *(MAP) reconciliations and realizations can be computed. Finally, we describe how the difference between two reconciliations can be quantified.

### The DLRS model and notation

**Figure 1:**
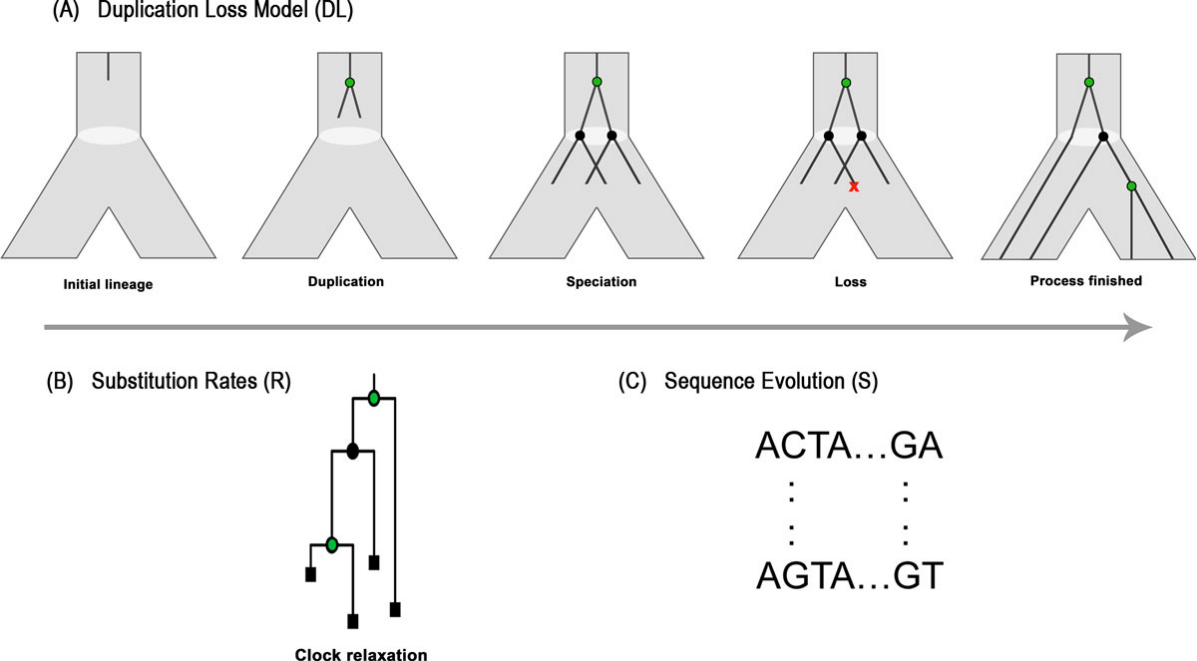
**The three submodels of DLRS are shown**. (A) Evolution of a gene lineage inside a species tree edge is modeled by a birth-death process. A gene lineage may come across a duplication event (represented by a green vertex), or a speciation event (represented by a black vertex). Every time a gene lineage passes through a speciation event, it splits into two independent gene lineages. A gene lineage may also be lost (represented by a red cross). After pruning all lost lineages, the final gene tree is obtained. (B) A relaxed molecular clock is employed to achieve branch lengths. (C) Finally, a standard sequence evolution model generates sequences over the gene tree with branch lengths.

The DLRS model, proposed by Åkerborg et. al. in [4], is based on three submodels: a duplication & loss model (DL), a substitution rate model (R), and a sequence evolution model (S) (see Figure 1). The duplication & loss submodel captures the evolution of a gene tree inside a species tree with given divergence times. For a tree *T*, we use *V *(*T*), *E*(*T*), and *L*(*T*) to denote the set of vertices, edges, and leaves of a tree *T*, respectively. Along an edge *e *∈ *E*(*S*) of the species tree, gene duplications and losses are modeled by a linear birth-death process. The duplication & loss process has two rates that are used, in the natural way, as rates for the birth-death process. A relaxed molecular clock is assumed for the substitution rate submodel. The gene tree edges have substitution rates, which are independently and identically Γ-distributed and parameterized by a mean and a variance. The sequence evolution submodel, can be any standard sequence evolution model, e.g., JTT, which is the case of this study.

We use planted binary gene and species trees, i.e., the trees can be obtained by adding a new vertex, a so-called planted root, to a rooted binary tree and making the planted root and the root adjacent. Moreover, let *θ *= (*λ, µ, m, v, M *) be the parameters of the model, where *λ *is the gene duplication rate, *µ *the gene loss rate, *m *the mean and *v *the variance of the distribution for sequence evolution rates across gene tree edges, and, finally, *M *the parameters of the sequence evolution model. Let *T *be a rooted tree and *u *∈ *V *(*T*). The subtree of *T *rooted at *u, T_u_*, is the minimal subtree of *T *containing all descendants of *u*, including *u*. The subtree of *T *planted at *u*, denoted *T^u ^*is defined to be the subtree rooted at *u, T_u_*, together with the edge from *u *to its parent.

### MCMC estimation of DLRS posterior over gene trees

We now describe the MCMC based framework employed in [4], which uses the Metropolis-Hastings algorithm for inference of the posterior over gene trees and rate parameters, and we also show how it can be extended to facilitate sampling of reconciliations from the posterior distribution. A state of the MCMC chain is a triple (*G, l, θ*) where the components are: a gene tree *G *with lengths *l*, and the parameters of the DLRS model *θ*. We use *P *(*·*) to denote a probability and *p*(*·*) to denote a probability density. Let (*G, l, θ*) be the current state and let (*G*', *l*', *θ*') be the proposed state in an iteration of the MCMC algorithm. The acceptance probability of the proposed state (*G*', *l*', *θ*') is determined by the ratio of the two probability densities *p*(*G, l, θ|D, S*) and *p*(*G*', *l*', *θ*'*|D, S*), where *D *is gene sequence data and *S *is the species tree. Since each such can be express using Bayes equality, e.g.,

$$p\left(G,l,\theta |D,S\right)=\frac{P\left(D|G,l\right)p\left(G,l|\theta ,S\right)p\left(\theta \right)}{P\left(D|S\right)},$$

the denominators cancel and we obtain

$$\frac{p\left(G,l,\theta |D,S\right)}{p\left(G^{\prime },l^{\prime },\theta^{\prime }|D,S\right)}=\frac{P\left(D|G,l\right)p\left(G,l|\theta ,S\right)p\left(\theta \right)}{P\left(D|G^{\prime },l^{\prime }\right)p\left(G^{\prime },l^{\prime }|\theta^{\prime },S\right)p\left(\theta^{\prime }\right)}.$$

Here the numerator and denominator have the same structure, so it is sufficient to describe how to compute the former. First, the factor *P *(*D|G, l*) can be computed using the dynamic programming (DP) algorithm proposed by Felsenstein [10]. Second, the prior *p*(*θ*) is chosen so that it can be easily computed. Finally, the main technical contribution of [4] is a DP algorithm for computing the remaining factor *p*(*G, l|θ, S*), and we continue by outlining that approach.

Let us first define some key concepts. Let *S*' be a discretized species tree where edges of the species tree *S *have been augmented with additional discretization vertices such that all the augmented vertices are equidistant within an edge, see supplementary Figure 2 in additional file 1.

Furthermore, we define a *reconciliation *to be a mapping of vertices of a gene tree *V *(*G*) to the vertices and edges of the species tree, i.e., *V *(*S*) ∪ *E*(*S*). A *discretized realization*, or *d-realization, α*, is a mapping of vertices of a gene tree *G *to the vertices of the discretized species tree *S*'.

A realization never maps a vertex and its parent to same vertex *x *∈ *V *(*S*'). We consider sound reconciliations and realizations, e.g., they never map a vertex of the gene tree *u *closer to the root than the position to which it maps its parental vertex, and ensures *G *is properly embedded within *S*. Moreover, let *σ*(*u*) be the function defined as follows (i) for a leaf *u *∈ *L*(*G*), *σ*(*u*) is the species tree leaf in which the gene that *u *represents can be found and (ii) for any internal vertex *u *of *G, σ*(*u*) is the least common ancestor of *L*(*G_u_*) in *S*.

### Extending the posterior to d-realizations or reconciliations

In order to extend the MCMC sampling from the posterior over gene trees with lengths and parameters, i.e., *p*(*G, l, θ|D, S*) to sampling also over d-realizations, i.e., *p*(*G, l, α, θ|D, S*), it is sufficient to be able to sample from *p*(*α|G, l, θ, S*). This conclusion follows from

$$p\left(G,l,\alpha ,\theta |D,S\right)=p\left(\alpha |G,l,\theta ,D,S\right)p\left(G,l,\theta |D,S\right)=p\left(\alpha |G,l,\theta ,S\right)p\left(G,l,\theta |D,S\right).$$

The analogous statement is true for a reconciliation, *γ*; that is, if we can sample from the reconciliation posterior distribution *p*(*γ|G, l, θ, S*), then we can also sample from the full posterior *p*(*G, l, γ, θ|D, S*).

In practice, sampling from the posterior extended with d-realizations, *p*(*G, l, α, θ|D, S*), is perfomed by first running the DLRS posterior MCMC so that *k *samples (*G*_1_, *l*_1_, *θ*_1_), ..., (*G_k_, l_k_, θ_k_*) are obtained and, then for each *i *∈ [*k*], sample *α_i _*from *p*(*α_i_|G_i_, l_i_, θ_i_, S*). The samples from *p*(*G, l, α, θ|D, S*) are, finally, (*G*_1_, *l*_1_, *α*_1_, *θ*_1_), ..., (*G_k_, l_k_, α_k_, θ_k_*).

There is a unique reconciliation associated with each realization and the posterior probability of a reconciliation is approximated by the sum of the posterior probabilities of the d-realizations associated with it. Thus, we can sample a reconciliation, from the posterior distribution over those, by sampling a d-realization, from the posterior over those, and then outputting the associated reconciliation, which easily can be computed. So by following the above described procedure and then, for each *i *∈ [*k*], computing the reconciliation *γ_i _*associated with *α_i_*, we obtain *k *samples (*G*_1_, *l*_1_, *γ*_1_, *θ*_1_), ..., (*G_k_, l_k_, γ_k_, θ_k_*) from the posterior distribution over reconciliations and other parameters.

### The generation probability and d-realization sampling

**Figure 2:**
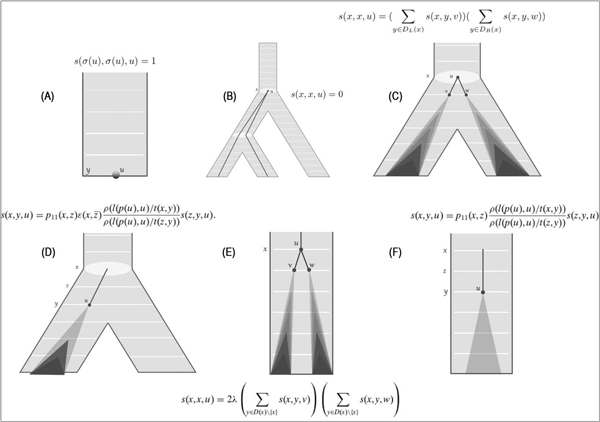
**Recursion to compute *p*(*G, l|θ, S*)**. Shows six possible scenarios of a gene tree evolving inside the species tree and how they relate to dynamic programming calculations. (A) A gene tree lineage starts and reaches *y*, where *y* is a leaf of an extant species. (B) An internal vertex of a gene tree cannot be mapped to a speciation vertex other than the least common ancestor of its children in species tree. (C) An internal vertex of a gene tree may be placed on a speciation node (the least common ancestor of its children in the species tree). (D) A gene subtree rooted at *u*, starts from *x *(a speciation vertex), duplicates at *y *and yields the subtree below. (E) A gene subtree rooted at *u *is placed at some discretization point on an edge of species tree. (F) A gene lineage starts from a discretization point on an edge of species tree, and yields the gene subtree rooted at *u*.

In [4], a DP algorithm for computing the factor *p*(*G, l|θ, S*) was described (Figure 2). The DP makes use of a table, *s*(*x, y, u*), defined as the probability that when a single gene lineage starts to evolve at the vertex *x *∈ *V *(*S*'), the tree *G^u ^*is generated together with the edge lengths *l *and, moreover, the event corresponding to *u *occurs at *y *∈ *V *(*S*'). Let *v *and *w *be children of *u *in *G*, and let *x, y, z *be vertices of *V *(*S*'). Let *ρ*(*r*) be the probability that an edge of *G *has rate *r*. Also, let *t*(*x, y*) be the time between vertices *x, y *∈ *V *(*S*'). The following recursions describe how the table *s *can be computed:

1. If *u *∈ *L*(*G*) and *x = σ*(*u*), *s*(*x, x, u*) = 1.

2. If *x *∈ *V *(*S*) and *x ≠ σ*(*u*), *s*(*x, x, u*) = 0.

3. If *x *∈ *V *(*S*)*\L*(*S*) and *x = σ*(*u*),

$$s\left(x,x,u\right)=\left(\underset{y\in D_{L}\left(x\right)}{\sum }s\left(x,y,v\right)\right)\left(\underset{y\in D_{R}\left(x\right)}{\sum }s\left(x,y,w\right)\right),$$

where *D_L_*(*x*) and *D_R_*(*x*) are the descendants of left and right child of *x *in *S*', respectively.

4. If *x *∈ *V *(*S*) and *z *is a child of *x *such that $\sigma \left(L\left(G_{u}\right)\right)\subseteq L\left(S_{z}^{\prime }\right)$and *z *is an ancestor of *y*,

$$s\left(x,y,u\right)=p_{11}\left(x,z\right)\varepsilon \left(x,\bar{z}\right)\frac{\rho \left(1\left(p\left(u\right),u\right)/t\left(x,y\right)\right)}{\rho \left(l\left(p\left(u\right),u\right)/t\left(z,y\right)\right)}s\left(z,y,u\right),$$

where $\varepsilon \left(x,\bar{z}\right)$ is the probability that a gene lineage starting at *x *does not reach any leaf $l\in L\left(S_{x}^{\prime }\right)\backslash L\left(S_{z}^{\prime }\right)$.

However, if *y = z*, the expression reduces to the following,

$$s\left(x,y,u\right)=p_{11}\left(x,y\right)\varepsilon \left(x,\bar{y}\right)\rho \left(l\left(p\left(u\right),u\right)/t\left(x,y\right)\right)s\left(y,y,u\right).$$

5. If *x *∈ *V *(*S*')*\V *(*S*),

$$s\left(x,x,u\right)=2\lambda \left(\underset{y\in D\left(x\right)\backslash \left\{x\right\}}{\sum }s\left(x,y,v\right)\right)\left(\underset{y\in D\left(x\right)\backslash \left\{x\right\}}{\sum }s\left(x,y,w\right)\right),$$

where *D*(*x*) is the set of descendants of *x*.

6. If *x *∈ *V *(*S*')*\V *(*S*) and *z *is the child of *x *in the discretized species tree *S*',

$$s\left(x,y,u\right)=p_{11}\left(x,z\right)\frac{\rho \left(l\left(p\left(u\right),u\right)/t\left(x,y\right)\right)}{\rho \left(l\left(p\left(u\right),u\right)/t\left(z,y\right)\right)}s\left(z,y,u\right).$$

However, if *y = z*, the expression reduces to the following,

$$s\left(x,y,u\right)=p_{11}\left(x,y\right)\rho \left(l\left(p\left(u\right),u\right)/t\left(x,y\right)\right)s\left(y,y,u\right).$$

In any reconciliation or d-realization, the planted root of *G *is mapped to the planted root of *S*. The probability that the gene tree *G *is generated is the probability that when a single lineage starts at the root of *S*, the root of *G *occurs somewhere below the planted root of *S *and then the process continues and generates *G*. Hence,

$$p\left(G,l|\theta ,S\right)=\underset{y\in D\left(p\right)}{\sum }s\left(p,y,r\right),$$

where *p *is the planted root of *S, D*(*p*) its descendants, and *r *is the root of *G*. Consequently, the probability that *r *is mapped to *y *∈ *V *(*S*') by a d-realization sampled from all d-realizations according to the posterior probability distribution under observed *G *and *l*, is

$$\frac{s\left(p,y,r\right)}{p\left(G,l|\theta ,S\right)}=\frac{s\left(p,y,r\right)}{\sum_{z\in D\left(p\right)}s\left(p,z,r\right)}.$$

Similarly, if we know that a d-realization maps a vertex *u *∈ *V *(*G*) to a vertex *x *∈ *V *(*S*'), then the probability that a child *v *of *u *is mapped to *y *by a realization sampled from all such d-realizations, according to the posterior probability distribution under observed *G *and *l*, is

$$\frac{s\left(x,y,u\right)}{\sum_{z\in D\left(x\right)}s\left(x,z,u\right)}.$$

This clearly provides an algorithm for sampling d-realizations according the posterior probability distribution under observed *G *and *l*.

Again, there is a unique reconciliation associated with each realization, and the posterior probability of a reconciliation is approximated by the sum of the posterior probabilities of the d-realizations associated with it. Thus, we can sample a reconciliation from the posterior distribution, over those, by sampling a d-realization, from the posterior over those, and then outputting the associated reconciliation.

### Computing the MAP d-realization

We now give recursions that imply a DP algorithm for computing the MAP reconciliation. The following recursions describe how *m *can be computed, and as apparent, they are very similar to those above for *s*:

1. If *u *∈ *L*(*G*) and *x = σ*(*u*), *m*(*x, x, u*) = 1.

2. If *x *∈ *V *(*S*) and *x ≠ σ*(*u*), *m*(*x, x, u*) = 0.

3. If *x *∈ *V *(*S*)*\L*(*S*) and *x = σ*(*u*),

$$m\left(x,x,u\right)=\left(\underset{y\in D_{L}\left(x\right)}{\text{max}}m\left(x,y,v\right)\right)\left(\underset{y\in D_{R}\left(x\right)}{\text{max}}m\left(x,y,w\right)\right),$$

where *D_L_*(*x*) and *D_R_*(*x*) are the descendants of left and right child of *x *in *S*', respectively.

4. If *x *∈ *V *(*S*) and *z *is a child of *x *such that $\sigma \left(L\left(G_{u}\right)\right)\subseteq L\left(S_{z}^{\prime }\right)$ and *z *is an ancestor of *y*,

$$m\left(x,y,u\right)=p_{11}\left(x,z\right)\varepsilon \left(x,\bar{z}\right)\frac{\rho \left(l\left(p\left(u\right),u\right)/t\left(x,y\right)\right)}{\rho \left(l\left(p\left(u\right),u\right)/t\left(z,y\right)\right)}m\left(z,y,u\right),$$

where $\varepsilon \left(x,\bar{z}\right)$ is the probability that a gene lineage starting at *x *does not reach any leaf $l\in L\left(S_{x}^{\prime }\right)\backslash L\left(S_{z}^{\prime }\right)$.

However, if *y = z*, the expression reduces to the following,

$$m\left(x,y,u\right)=p_{11}\left(x,y\right)\varepsilon \left(x,\bar{y}\right)\rho \left(l\left(p\left(u\right),u\right)/t\left(x,y\right)\right)m\left(y,y,u\right).$$

5. If *x *∈ *V *(*S*')*\V *(*S*),

$$m\left(x,x,u\right)=2\lambda \left(\underset{y\in D\left(x\right)\backslash \left\{x\right\}}{\text{max}}m\left(x,y,v\right)\right)\left(\underset{y\in D\left(x\right)\backslash \left\{x\right\}}{\text{max}}m\left(x,y,w\right)\right),$$

where *D*(*x*) is the set of descendants of *x*.

6. If *x *∈ *V *(*S*')*\V *(*S*) and *z *is the child of *x *in the discretized species tree *S*',

$$m\left(x,y,u\right)=p_{11}\left(x,z\right)\frac{\rho \left(l\left(p\left(u\right),u\right)/t\left(x,y\right)\right)}{\rho \left(l\left(p\left(u\right),u\right)/t\left(z,y\right)\right)}m\left(z,y,u\right).$$

However, if *y = z*, the expression reduces to the following,

$$m\left(x,y,u\right)=p_{11}\left(x,y\right)\rho \left(l\left(p\left(u\right),u\right)/t\left(x,y\right)\right)m\left(y,y,u\right).$$

We now get an expression for the probability of the MAP d-realizations, very similar to that of *p*(*G, l|θ, S*),

$$\underset{\alpha }{\text{max}}\frac{p\left(G,l,\alpha |\theta ,S\right)}{p\left(G,l|\theta ,S\right)}=\underset{y\in D\left(p\right)}{\text{max}}\frac{m\left(p,y,r\right)}{p\left(G,l|\theta ,S\right)}.$$

When computing the probability of the MAP d-realizations, we can use the standard technique of back-pointers, i.e., keep track of the subsolution that gives the maximum value, and after the computation of *m*, trace the backpointers in order to find a MAP d-realization.

### Posterior probability of a given reconciliation

We now give recursions for computing the posterior probability of a given reconciliation *γ*, i.e., *p*(*G, l, γ|θ, S*). The reconciliation is a mapping from *V *(*G*) to *V *(*S*) ∪ *E*(*S*). Let *R *be the function from *V *(*S*) ∪ *E*(*S*) to *V *(*S*') defined by (i) for *x *∈ *V *(*S*), *R*(*x*) = *x *and (ii) for (*x, y*) ∈ *E*(*S*), *R*(*x, y*) is the set of internal vertices on the unique path between *x *and *y *in *S*'.

1. If *u *∈ *L*(*G*) and *x = γ*(*u*), *s*(*x, x, u*) = 1.

2. If *x *∈ *V *(*S*) and *x ≠ γ*(*u*), *s*(*x, x, u*) = 0.

3. If *x *∈ *V *(*S*)*\L*(*S*) and *x = γ*(*u*),

$$s\left(x,x,u\right)=\left(\underset{y\in D_{L}\left(x\right)\cap R\left(\gamma \left(v\right)\right)}{\sum }s\left(x,y,v\right)\right)\left(\underset{y\in D_{R}\left(x\right)\cap R\left(\gamma \left(w\right)\right)}{\sum }s\left(x,y,w\right)\right),$$

where *D_L_*(*x*) and *D_R_*(*x*) are the descendants of left and right child of *x *in *S*', respectively.

4. If *x *∈ *V *(*S*) and *z *is a child of *x *such that $\sigma \left(L\left(G_{u}\right)\right)\subseteq L\left(S_{z}^{\prime }\right)$ and *z *is an ancestor of *y*,

$$s\left(x,y,u\right)=p_{11}\left(x,z\right)\varepsilon \left(x,\bar{z}\right)\frac{\rho \left(l\left(p\left(u\right),u\right)/t\left(x,y\right)\right)}{\rho \left(l\left(p\left(u\right),u\right)/t\left(z,y\right)\right)}s\left(z,y,u\right),$$

where $\varepsilon \left(x,\bar{z}\right)$ is the probability that a gene lineage starting at *x *does not reach any leaf $l\in L\left(S_{x}^{\prime }\right)\backslash L\left(S_{z}^{\prime }\right)$.

However, if *y = z*, the expression reduces to the following,

$$s\left(x,y,u\right)=p_{11}\left(x,y\right)\varepsilon \left(x,\bar{y}\right)\rho \left(l\left(p\left(u\right),u\right)/t\left(x,y\right)\right)s\left(y,y,u\right).$$

5. If *x *∈ *V *(*S*')*\V *(*S*),

$$s\left(x,x,u\right)=2\lambda \left(\underset{y\in (D\left(x\right)\cap R\left(\gamma \left(v)\right)\right)\backslash \{x\}}{\sum }s\left(x,y,v\right)\right)\left(\underset{y\in (D\left(x\right)\cap R(\gamma \left(w)\right))\backslash \{x\}}{\sum }s\left(x,y,w\right)\right),$$

where *D*(*x*) is the set of descendants of *x*.

6. If *x *∈ *V *(*S*')*\V *(*S*) and *z *is the child of *x *in the discretized species tree *S*',

$$s\left(x,y,u\right)=p_{11}\left(x,z\right)\frac{\rho \left(l\left(p\left(u\right),u\right)/t\left(x,y\right)\right)}{\rho \left(l\left(p\left(u\right),u\right)/t\left(z,y\right)\right)}s\left(z,y,u\right).$$

However, if *y = z*, the expression reduces to the following,

$$s\left(x,y,u\right)=p_{11}\left(x,y\right)\rho \left(l\left(p\left(u\right),u\right)/t\left(x,y\right)\right)s\left(y,y,u\right).$$

Finally,

$$p\left(G,l,\gamma |\theta ,S\right)=\underset{y\in D\left(p\right)\cap R\left(\gamma \left(r\right)\right)}{\sum }s\left(p,y,r\right),$$

where *p *is the planted root of *S, D*(*p*) its descendants, and *r *is the root of *G*.

### Comparing reconciliations

**Figure 3:**
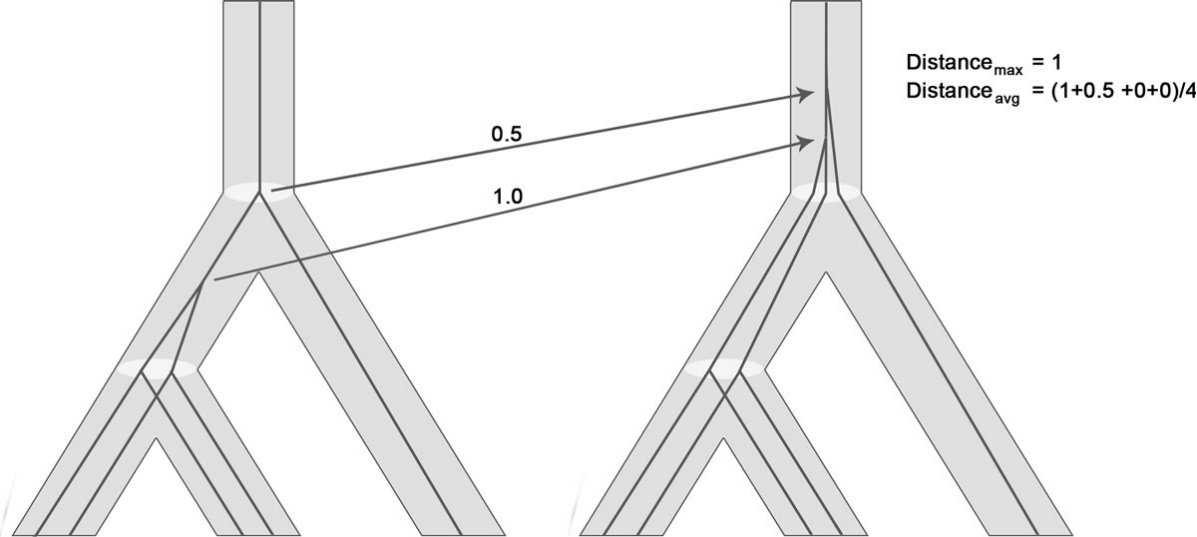
**Computing distance between two reconciliations**. Illustrates the computation of maximum distance and average distance between two reconciliations that differ in the placement of two vertices.

We are interested in quantifying the difference between two reconciliations *γ *and *γ*' of *G *and *S*, in particular between a reconciliation we have sampled from the posterior and the MPR. To this end, we introduce two distance measures. First, however, an atomary distance between objects in *V *(*S*) ∪ *E*(*S*) is defined, so that for any vertex *u *∈ *V *(*G*), the distance between *γ*(*u*) and *γ*'(*u*) is well-defined. We can then compute the two reconciliation distance measures, namely (i) the maximum atomary distance over vertices of *G*, and (ii) the average atomary distance over vertices of *G *(Figure 3).

Assume that *a, b *∈ *V *(*S*) ∪ *E*(*S*). Let *l *be the length of the minimum length path of *S *that contains both *a *and *b*, and let *d*(*a, b*) = *l *+ 1 *− |*{*a, b*} ∩ *V *(*S*)*|/*2 *− |*{*a, b*} ∩ *E*(*S*)*|*. So, for instance, if *a *is a vertex and *b *is the edge to the parent of *a*, then *d*(*a, b*) = 1 + 1 *− *1*/*2 *− *1 = 0.5, and if *a *= (*x, p_S _*(*x*)) (where *p_S _*(*·*) denotes the parent function in *S*) and *b *= (*p_S _*(*x*), *p_S _*(*p_S _*(*x*)), then *d*(*a, b*) = 2 + 1 *− *1 *− *1 = 1. We are now ready to define our distances between reconciliations: the max distance is

$$distance_{max}\left(\gamma ,\gamma^{\prime }\right)=max_{u\in V\left(G\right)}d\left(\gamma \left(u\right),\gamma^{\prime }\left(u\right)\right),$$

and the average distance is

$$distance_{avg}\left(\gamma ,\gamma^{\prime }\right)=\frac{\sum_{u\in V\left(G\right)}d\left(\gamma \left(u\right),\gamma^{\prime }\left(u\right)\right)}{|V\left(G\right)\backslash L\left(G\right)|}.$$

### Data

See Supplementary Material & methods (additional file 1).

## Results

We applied our methods to the vertebrates clade of the OPTIC dataset, [9], consisting of the following nine vertebrate species: *Tetraodon nigroviridis *(pufferfish), *Monodelphis domestica *(gray short-tailed opossum), *Canis familiaris *(dog), *Mus musculus *(house mouse), *Homo sapiens *(human), *Ornithorhynchus anatinus *(platypus), *Taeniopygia guttata *(zebra finch), *Gallus gallus *(red junglefowl), and *Anolis carolinensis *(carolina anole). After basic filtering, 13812 gene families were selected for analysis, see supplementary Material and methods in additional file 1.

For each gene family, using the MCMC-based analysis tool PrIME-DLRS [4][11], a posterior distribution was obtained over gene trees, edge lengths and other parameters, given gene sequences and the species tree. The expected number of duplications under the posterior distribution, given the gene families and the species tree, was then estimated by sampling d-realizations and recording the number of duplications occurring at any specific discretization vertex. The number of duplications for all discretization vertices of the species tree were then normalized to 11 levels and each level was assigned a specific color. So, the colored heatmap illustrates how frequent duplications have been across the species tree. We also investigated enrichment of functional categories among the gene families with higher expected number of duplications over an edge. Finally, the appropriateness of MPR was investigated, by estimating the expected average and maximum distance, respectively, to MPR over reconciliations sampled from the posterior; a few families for which MPR was found to be unsuitable were analyzed further.

### Heatmaps

**Figure 4:**
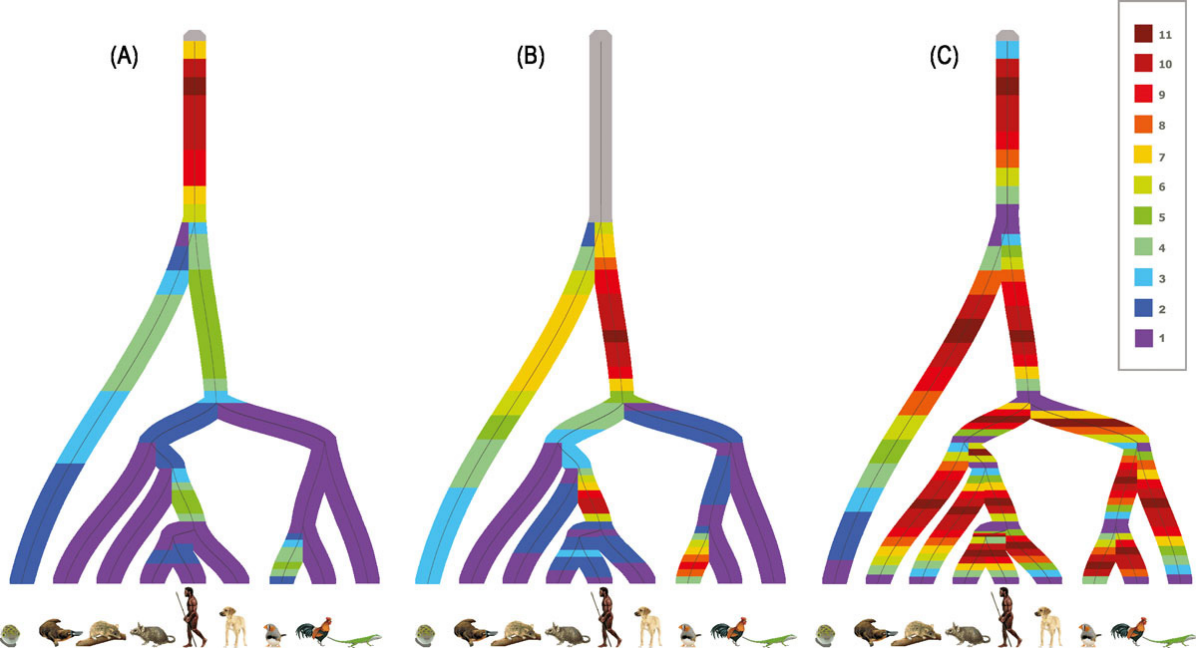
**Heatmaps of duplications across the discretized edges of the species tree**. (A) The heatmap is generated after normalizing the duplications across the tree using eleven different colors. (B) The heatmap is generated after normalizing the duplications across the tree without including the common ancestral edge. (C) The heatmap is generated by normalizing the duplications for each edge of the species tree.

Heatmaps of the number of the duplications for the posterior distribution over realizations were generated, and provide a visualization of the duplication patterns across the edges of the species tree, Figure 4A. The highest number of duplications were observed at the common ancestral edge of all the species. This could be interpreted as support of the 2R hypothesis proposed by Ohno [12], which suggests that the genome of early vertebrates underwent two whole genome duplications. An alternative explanation could be that incorrect gene trees in the posterior distributions give rise to duplication that reconciliations tend to place close to the planted root. In order to test the latter, we performed a *Maximum aposteriori *(MAP) analysis based on only gene families with MAP gene trees having posterior probability grather than or equal to 0.5. Heatmaps based on this data, supplementary Figure 1 (supplementary results in additional file 1), showed the same trend.

The common ancestral edge of all the species except Puffer Fish had the second highest number of duplications among all the edges of the species tree as shown in Figure 4A. In order to study the more recent lineages more closely, we normalized the duplications across the species tree without the discretization points of common ancestral edge, see Figure 4B. As the figure shows, the common ancestral edge of Human, Dog, and Mouse as well as the edge leading to Zebra Finch have comparatively higher frequencies of duplications. The higher frequencies of duplication on the edge leading to Boreoeutheria (ancestral edge of Human, Dog and Mouse) was also reported recently by Boussau et al [13].

We decided to explore the families that contribute to these duplications. Tools for performing enrichment analysis allows analysis of extant but not ancestral species and, moreover, when studying duplicating genes, choosing representative genes in extant species is complicated by the fact that the number of representatives can be varied. We, therefore, decided to work with gene families, rather than genes, and implemented this by using a single representative gene for each family. We selected the representative gene for an edge if its gene family were found to be likely to duplicate at least once on the edge. This set of genes was then annotated using the Functional Annotation Clustering (DAVID) [14,15] tool given the background of all the representative genes of the gene families of the dataset. For the common ancestral edge of Human, Dog, and Mouse, the following clusters had a Benjamini-Hochberg false discovery rate less than or equal to 0.01: *ATP Binding, Chromosome Segregation/Mitosis, ATPase activity coupled to movement of substances, Drug Metabolism, Helicase Activity, Mitotic Sister Chromatid Segregation, Fatty Acid Metabolism/Tryptophan Metabolism *and *Death-like Domain*. Using the same criteria for the ancestral edge of Zebra Finch, gave the following clusters: *Transit Peptide, Mitochondrion, ATP Binding, Flavoprotein *and *Nucleotide Phosphatebinding region*.

In order to know how the frequency of duplications vary across each edge of the species tree, the heatmap was also normalized across edges (see Figure 4C). In most cases, there is a unimodal behavior of an individual edge. This may be explained by the relatively low number of discretization vertices and also that the signal in the sequence data may not be strong enough to reveal a more complex trend. For individual gene families, however, more complex trends are exhibited.

### Distance from MPR

**Figure 5:**
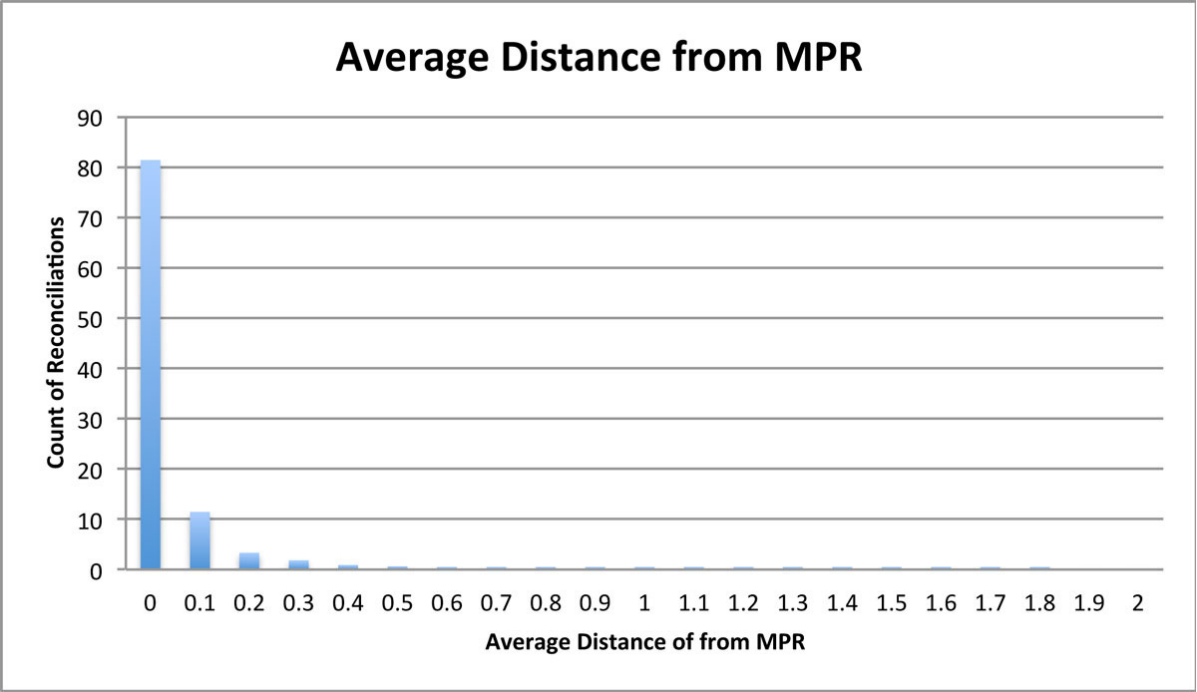
**Average distance from MPR across gene families**. X-axis represents average distance of a reconciliation from the MPR. Y-axis represents the percentage of count of reconciliations having a certain average distance from the MPR

We computed two distances, i.e., the average distance and maximum distance from MPR, over the posterior distribution. The distribution of average distance between the sampled reconciliations and the MPR is shown in the Figure 5. The MPRs dominates the distribution of sampled reconciliations with approximately 81% of all sampled reconciliations. We computed the expected distance for posterior reconciliations of individual gene families to MPR, in order to identify gene families with a clear signal for early duplications, i.e., early in the sense of being inferred as significantly earlier than according to MPR.

**Figure 6:**
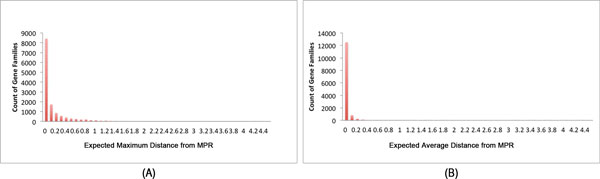
**Expected distances from the MPR for the 13812 analyzed gene families**. (A) Distribution of the expected maximum distance to MPR. (B) Distribution of the expected average distance to MPR..

The expected distance of a family was estimated as the expected average distance to MPR over reconciliations sampled from the posterior. The results showed that a number of gene families have a higher expected distance from the MPR, which means that MPR does not explain the true evolutionary history well in those cases. About 13% of all families had expected maximum distance equal to or greater than 0.5. The distribution of the expected maximum distance and the expected average distance of the gene families from the MPR are shown in Figure 6.

**Figure 7:**
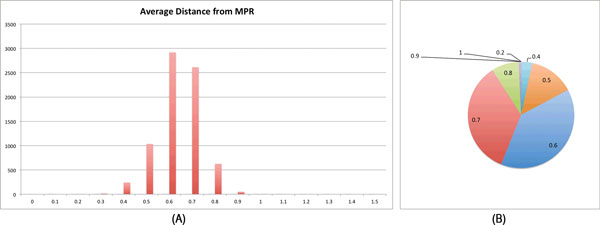
**Average distance from MPR for the gene family *Short chain dehydrogenase***. (A) Distribution of the average distance to MPR over 10000 sampled reconciliations. (B) The pie chart shows the shares of reconciliations having a certain average distance from the MPR. The labels shows the distance from the MPR.

We selected four gene families for further analysis. They had a clear signal for early duplications and at least one gene from every species of the dataset. One of the four selected gene families was *Short chain dehydrogenase*. It has a clear signal in favor of non-MPR reconciliations as shown in Figure 7. Most of the reconciliations sampled for this family had average distances between 0.5 and 0.6, comprising around 74% of all sampled reconciliations. For this gene family, not a single reconciliation sampled was identical to the MPR. This gene family is annotated as steroid hormone biosynthesis.

## Conclusion

We have presented methods for sampling and computing MAP reconciliations as well as d-realizations. Using these methods and the OPTIC dataset, we have provided the first biologically realistic estimate of the appropriateness of MPR. It was found that one can expect approximately 19% of reconciliations to be different from MPR. Also, 13% of gene families can be expected to have a maximum distance of greater than or equal to 0.5 to the MPR. Among other reasons, this is interesting because some gene tree reconstuction algorithms evaluate gene trees using only MPR. We have also shown how, based on our methods, heatmaps can be constructed that illustrates how frequent duplications are across the species tree and that for vertebrates such a strategy identifies two recent edges as having hosted frequent duplications. Finally, enrichment analysis can identify functional classes among gene families that are duplicated on a specific species tree edge.

## Competing interests

The authors declare that they have no competing interests.

## Supplementary Material

Additional file 1 (PDF) Click here for file

## References

[B1] Goodman M Czelusniak J Moore GW Romero-Herrera A Matsuda G Fitting the gene lineage into its species lineage, a parsimony strategy illustrated by cladograms constructed from globin sequences Systematic Biology 1979 28 2 132 163 10.1093/sysbio/28.2.132

[B2] Arvestad L Berglund AC Lagergren J Sennblad B Bayesian gene/species tree reconciliation and orthology analysis using MCMC Bioinformatics 2003 19 suppl 1 i7 i15 10.1093/bioinformatics/btg1000 12855432

[B3] Arvestad L Berglund AC Lagergren J Sennblad B Gene tree reconstruction and orthology analysis based on an integrated model for duplications and sequence evolution Proceedings of the eighth annual international conference on Resaerch in computational molecular biology 2004 ACM 326 335

[B4] Åkerborg Ö Sennblad B Arvestad L Lagergren J Simultaneous Bayesian gene tree reconstruction and reconciliation analysis Proceedings of the National Academy of Sciences 2009 106 14 5714 5719 10.1073/pnas.0806251106 PMC2667006 19299507

[B5] Rasmussen MD Kellis M A Bayesian approach for fast and accurate gene tree reconstruction Molecular Biology and Evolution 2011 28 273 290 10.1093/molbev/msq189 20660489 PMC3002250

[B6] Hahn MW De Bie T Stajich JE Nguyen C Cristianini N Estimating the tempo and mode of gene family evolution from comparative genomic data Genome Research 2005 15 8 1153 1160 10.1101/gr.3567505 16077014 PMC1182228

[B7] Doyon JP Chauve C Hamel S Space of gene/species trees reconciliations and parsimonious models Journal of Computational Biology 2009 16 10 1399 1418 10.1089/cmb.2009.0095 19754270

[B8] Doyon JP Hamel S Chauve C An efficient method for exploring the space of gene tree/species tree reconciliations in a probabilistic framework Computational Biology and Bioinformatics, IEEE/ACM Transactions on 2012 9 26 39 10.1109/TCBB.2011.64 21464510

[B9] Heger A Ponting CP OPTIC: orthologous and paralogous transcripts in clades Nucleic acids research 2008 36 suppl 1 D267 D270 17933761 10.1093/nar/gkm852 PMC2238935

[B10] Felsenstein J Evolutionary trees from DNA sequences: a maximum likelihood approach Journal of molecular evolution 1981 17 6 368 376 10.1007/BF01734359 7288891

[B11] Sjöstrand J Sennblad B Arvestad L Lagergren J DLRS: gene tree evolution in light of a species tree Bioinformatics 2012 28 22 2994 2995 10.1093/bioinformatics/bts548 22982573

[B12] Ohno S Evolution by gene duplication 1970 London: George Alien & Unwin Ltd. Berlin, Heidelberg and New York: Springer-Verlag

[B13] Boussau B Szöllősi GJ Duret L Gouy M Tannier E Daubin V Genome-scale coestimation of species and gene trees Genome research 2013 23 2 323 330 10.1101/gr.141978.112 23132911 PMC3561873

[B14] Sherman BT Lempicki RA Bioinformatics enrichment tools: paths toward the comprehensive functional analysis of large gene lists Nucleic acids research 2009 37 1 13 10.1093/nar/gkn923 19033363 PMC2615629

[B15] Da Wei Huang BTS Lempicki RA Systematic and integrative analysis of large gene lists using DAVID bioinformatics resources Nature protocols 2008 4 44 57 10.1038/nprot.2008.211 19131956

