# Supplementary material for: Genome-wide probabilistic reconciliation analysis across vertebrates
Source: BMC Bioinformatics. 2013 Oct 15;14(Suppl 15):S10. doi: 10.1186/1471-2105-14-S15-S10 (PMC3852046; doi:10.1186/1471-2105-14-S15-S10)
Supplement: Additional file 1 — (PDF) [file 1471-2105-14-S15-S10-S1.PDF]

Supplementary materials
Materials & methods
Biological test data

The methods described in the main text Material & methods section are applied to the vertebrates dataset of OPTIC [1] consisting of nine species. The dataset was downloaded from http://genserv.anat.ox.ac.uk/downloads/clades/ and after basic filtering, 13812 gene families were selected for analysis. Uninformative positions were filtered out from the multiple sequence alignments provided by OPTIC using trimAl v. 1.2 with setting **-automated1** [2]. An analysis of suitable protein substitution models with ProtTest v. 3.2 [3] revealed that a majority of families favored the JTT model [4] with 4 gamma site rate categories [5] under the AICc criterion, while typically behaving well also among families reporting a different best model. This substitution model was used in all subsequent analyses. To acquire a high quality molecular dating of the vertebrate species tree, we extracted a subset of 200 families that (i) showed signs of conserved synteny using a simple triangular kernel scoring function on results obtained with CINTENY [6] and (ii) all favored the JTT protein substitution model [4]. These families are highly likely to be orthologous and to behave consistently. The multiple sequence alignments were concatenated, and then used to estimate relative divergence times using MAP-DP [7]. We selected the top *maximum a posteriori* (MAP) dating out of 70 MAP-DP runs, assuming a birth-death model for divergences, *iid* gamma edge rates, and the JTT substitution model with 4 gamma site rate categories. MAP-DP allows for rate variation across lineages as well as sites, and uses a molecular clock relaxation technique that corresponds to that used in the subsequent gene tree inference step; this can be expected to increase fit over estimates obtained by other means. JPriME, an MCMC-based analysis tool was employed to obtain the posterior distribution over gene trees, reconciliations and realizations, given gene sequences, and the species tree.

Results
MAP analysis of heatmaps

The heatmap analysis was repeated on a *maximum a posteriori* (MAP) gene tree subset of the posterior distribution obtained over realizations to avoid unwanted noise in the heatmaps. Gene families were only selected if the MAP gene tree was repeated in more than 50% of the posterior. The heatmaps were again generated(see figure 1), and they were more or less similar to the earlier heatmaps. We found no general pattern of duplications across the edges in this case either.

Four gene families with higher expected distance from the MPR

The four selected gene families with higher expected distance are "*Short chain dehydrogenase*", annotated as steroid hormone biosynthesis (KEGG Pathway), "*Peroxisomal trans-2-enoyl-CoA reductase*", annotated as biosynthesis of unsaturated fatty acids (KEGG Pathway), "*Arylacetamide deacetylase*", annotated as lipid metabolism (COG Ontology), and "*FYVE, RhoGEF and PH domain containing 3*", annotated as regulation of actin cytoskeleton (KEGG Pathway). *Short chain dehydrogenase* is discussed in the main text. *Peroxisomal trans-2-enoyl-CoA reductase* showed little support for MPR and in most cases favored non-MPR reconciliations (figure 3). Only 4% of the reconciliations sampled for the gene family were the MPRs. A significant portion (65%) of the reconciliations sampled had average distances between 0.5 and 0.7 from the MPR. *Arylacetamide deacetylase* also favored non-MPR reconciliations (figure 4), 81% of the reconciliations sampled had average distances between 0.4 and 0.6. *FYVE, RhoGEF and PH domain containing 3*, dealing with regulation of the actin cytoskeleton, is another such family showing strong support for non-MPR reconciliations (figure 5). Around 71% of the reconciliations sampled for this gene family had an average distances of 0.5 or 0.6 to the MPR.

References

1. Heger A, Ponting CP: **OPTIC: orthologous and paralogous transcripts in clades**. *Nucleic acids research* 2008, **36**(suppl 1):D267–D270.
2. Capella-Gutiérrez S, Silla-Martínez JM, Gabaldón T: **trimAl: a tool for automated alignment trimming in large-scale phylogenetic analyses**. *Bioinformatics* 2009, **25**(15):1972–1973.
3. Abascal F, Zardoya R, Posada D: **ProtTest: selection of best-fit models of protein evolution**. *Bioinformatics* 2005, **21**(9):2104–2105.
4. Whelan S, Goldman N: **A general empirical model of protein evolution derived from multiple protein families using a maximum-likelihood approach**. *Molecular biology and evolution* 2001, **18**(5):691–699.
5. Yang Z: **Maximum likelihood phylogenetic estimation from DNA sequences with variable rates over sites: approximate methods**. *Journal of Molecular evolution* 1994, **39**(3):306–314.
6. Sinha AU, Meller J: **Cinteny: flexible analysis and visualization of synteny and genome rearrangements in multiple organisms**. *BMC bioinformatics* 2007, **8**:82.
7. Åkerborg Ö, Sennblad B, Lagergren J: **Birth-death prior on phylogeny and speed dating**. *BMC Evolutionary Biology* **8**.

Supplementary figures

Supplementary figure 1

Heatmaps of duplications across the discretized edges of the species tree (MAP analysis). (A) Heatmap generated after normalizing the duplications across the tree using eleven different colors. (B) Heatmap generated after normalizing the duplications across the tree without the common ancestral edge. (C) Heatmap

generated by normalizing the duplications for each branch. These heatmaps were generated on only those gene families that had MAP gene trees in at least 50% of the posterior.

Supplementary figure 2

Reconciliations and realizations are illustrated. Each reconciliation have many compatible realizations. Black vertices of the gene tree are speciations, while the green vertices represent duplications. Discretization vertices are illustrated by white horizontal lines.

Supplementary figure 3

Average distance from MPR for the gene family *Peroxisomal trans-2-enoyl-CoA reductase.* (A) Distribution of the average distance to MPR over 10000 sampled reconciliations. (B) The pie chart shows the shares of reconciliations having a certain average distance from the MPR. The labels shows the distance from the MPR.

Supplementary figure 4

Average distance from MPR for the gene family *Arylacetamide deacetylase.* (A) Distribution of the average distance to MPR over 10000 sampled reconciliations. (B) The pie chart shows the shares of reconciliations having a certain average distance from the MPR. The labels shows the distance from the MPR.

Supplementary figure 5

Average distance from MPR for the gene family *FYVE, RhoGEF and PH domain containing 3.* (A) Distribution of the average distance to MPR over 10000 sampled reconciliations. (B) The pie chart shows the shares of reconciliations having a certain average distance from the MPR. The labels shows the distance from the MPR.

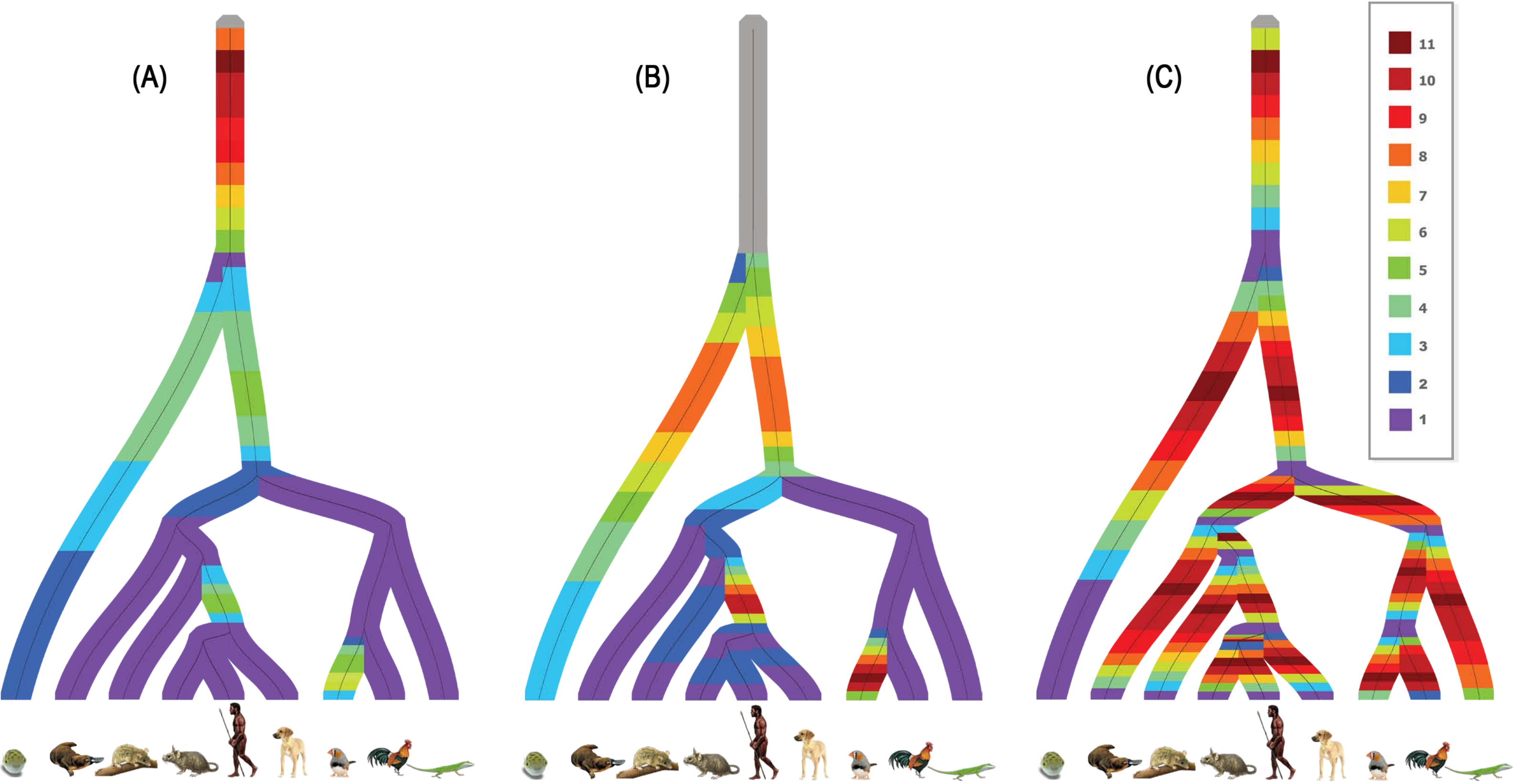
(A)
(B)
(C)
11
10
9
8
7
6
5
4
3
2
1

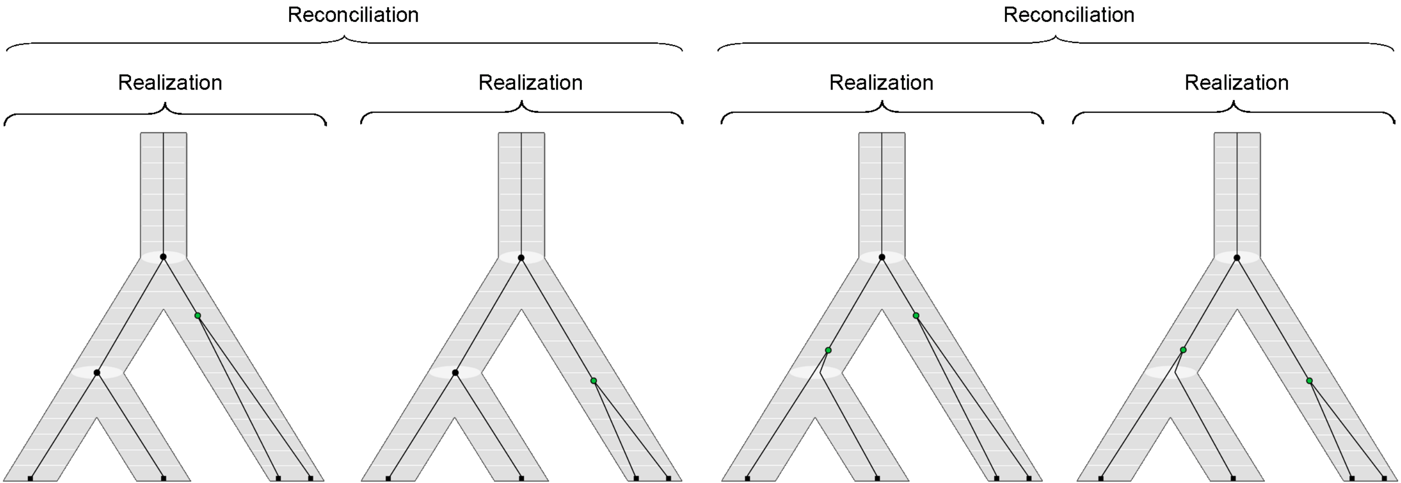

Reconciliation
Reconciliation
Realization
Realization
Realization
Realization

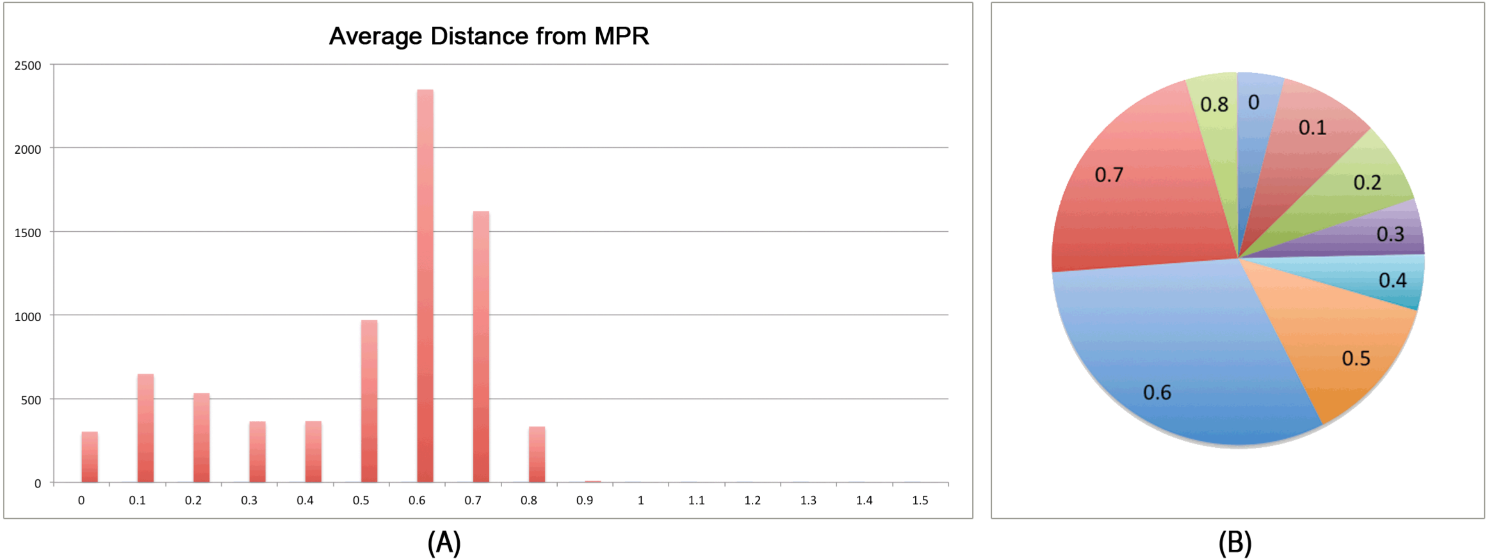
Average Distance from MPR
2500
2000
1500
1000
500
0
0
0.1
0.2
0.3
0.4
0.5
0.6
0.7
0.8
0.9
1
1.1
1.2
1.3
1.4
1.5
0.8
0
0.1
0.2
0.3
0.4
0.5
0.6
0.7
(A)
(B)

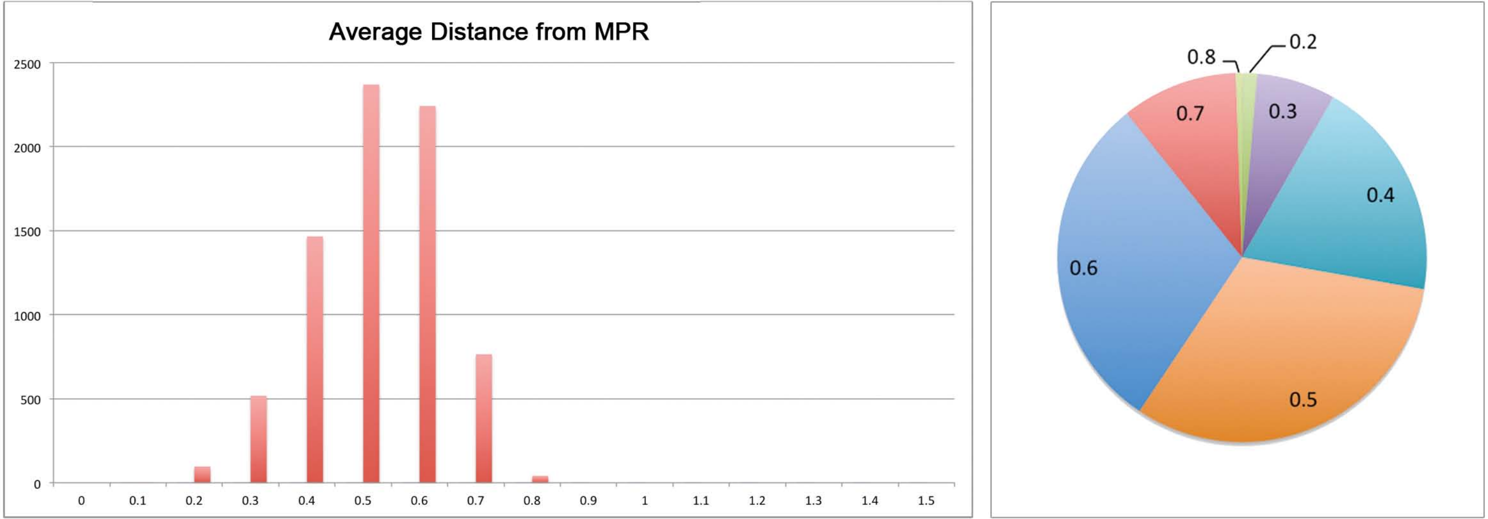
Average Distance from MPR
2500
2000
1500
1000
500
0
0
0.1
0.2
0.3
0.4
0.5
0.6
0.7
0.8
0.9
1
1.1
1.2
1.3
1.4
1.5
0.8
0.2
0.7
0.3
0.4
0.6
0.5
(A) (B)

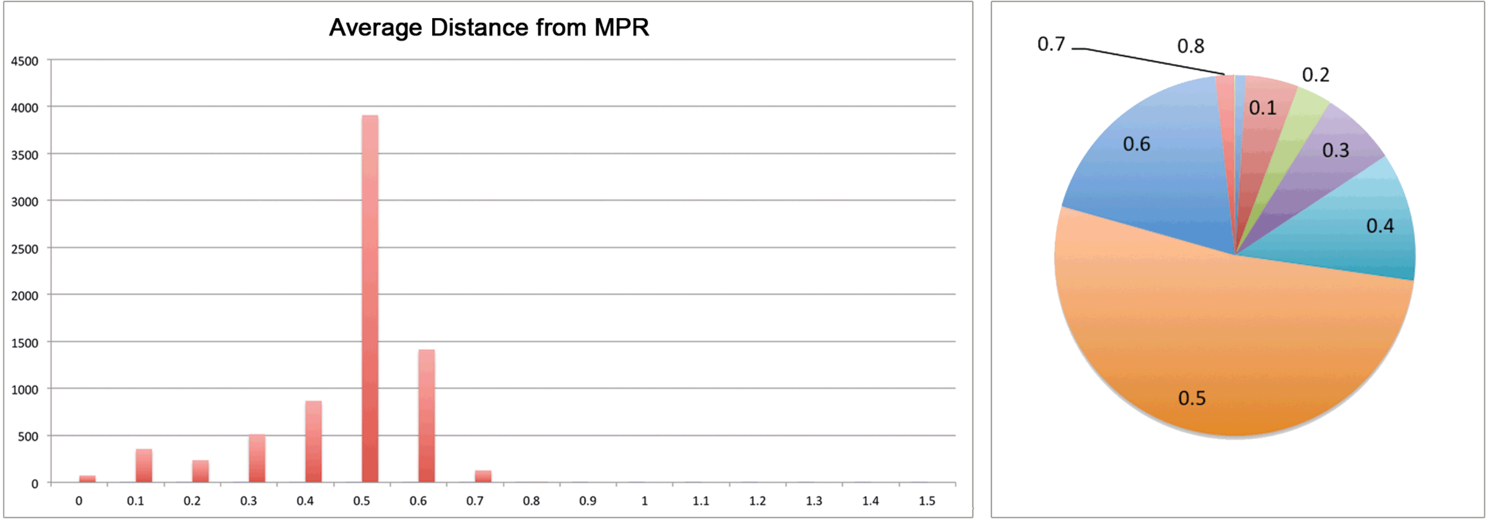
Average Distance from MPR
4500
4000
3500
3000
2500
2000
1500
1000
500
0
0
0.1
0.2
0.3
0.4
0.5
0.6
0.7
0.8
0.9
1
1.1
1.2
1.3
1.4
1.5
0.7
0.8
0.2
0.1
0.6
0.3
0.4
0.5

(A) (B)
